# Ambient x-ray pollution assessment at inspection gates of airports- a case study of Mehrabad and Imam Khomeini Airports in Iran

**DOI:** 10.1186/2052-336X-12-88

**Published:** 2014-05-28

**Authors:** Gholamhossein Pourtaghi, Firouz Valipour, Sepideh Nourian, Amirabbas Mofidi

**Affiliations:** 1 Health Research Center, Baqiyatallah University of Medical Sciences, Tehran, Iran; 2 Department of Occupational Health, Faculty of Health, Baqiyatallah University of Medical Sciences, Tehran, Iran; 3 Department of Occupational Health, School of Public Health, Shahid Beheshti University of Medical Science, Tehran, Iran; 4 Department of Occupational Health, Faculty of Medical sciences, Tarbiat Modares University, Tehran, Iran

**Keywords:** X-ray, Electromagnetic ionizing rays, Airport, Inspection gates

## Abstract

**Background:**

As a well-known, physical carcinogen, ambient X-ray pollution assessment would be of great importance in today’s modern world. Accordingly, the present study was done to measure the exposure level of ambient X-ray at inspection gates of two airports in Iran. According to which, the X-ray was measured at different points of the inspection gates including closed and opened Curtain, as well as seating place of operators beside the X-ray inspection systems. The recorded data were then analyzed by “sign” and t-tests.

**Results:**

The total average exposure level of the measured x-ray was 2.68 ± 0.73 μsv.h^-1^. The measured x-ray exposure level was 2.07 ± 0.61 (μsv.h^-1^) released from RAPISCAN X-ray inspection system and 3.3 ± 1.34 (μsv.h^-1^) emitted from HEIMANN X-ray inspection system. Comparison of average x-ray doses of the systems in both airports showed that the minimum and maximum exposure levels were recorded at 1(m) far from the devices and at the entrance of the devices, respectively.

**Conclusions:**

The exposure levels at all measurement points were lower than the occupational exposure limit. This reveals the fact that the exposed operators are not probably at risk of adverse health effects.

## Introduction

New technologies expose humans to various types of radiation [1,2]. X-rays are energetic electromagnetic radiations, which can ionize materials by ejecting electrons from atoms. It can cause cancer in exposed individuals and possibly impose harmful genetic mutations in their progeny. The extent of the ionization, absorption and molecular change depends on the quality (distribution of photon energy) and quantity (radiation intensity) of radiation. Living organisms that have exposed to ionizing radiation can be damaged or even die due to severe exposure. Cancer induction is one of the most important somatic risks of low dose ionizing radiation [3].

The study of Amy and Sarah showed about 0.6% cancer risk in those aged 75 years in UK and 0.6% to 1.8% censer risk in Japan as a result of exposure to diagnostic X-rays [4]. Delia et al. investigated occupational exposure in airport personnel to study the genotoxic and oxidative effects of x-ray. They found that the exposed group have a high mean value of sister chromatid exchange frequency and total structural chromosomal aberrations at particular breaks [5]. The most important characteristic of x-ray is its high penetration and ionization power. Easily passing through solid and liquid media it is used in radiography of different body organs. The x-ray is also used in radiography of metals to detect defected and fractured metal parts [6].

Nowadays, the use of imaging technologies releasing ionizing radiation for security control of goods, vehicles and persons have been the center of attention [7]. In 2009, Boeing Company estimated that there are 49,000 daily commercial flights around the world [8]. Statistics show that each year 107 pieces of luggage are screened at a large international airport [9]. This number clearly indicates that there is a great demand for security check of passengers’ luggage mainly to avoid smuggling or transporting illegal goods as well as fraud and terrorist threatening. X-ray detection, as the most common way for baggage screening in airports, provides a useful tool for inspecting baggage [10] by which it would be possible to check the content of packages without any damages [11]. There are various x-ray detection techniques, which facilitate inspecting luggage characteristics such as density and effective atomic number [12]. Theoretically, the material type of an object can uniquely be determined by two parameters of density and effective atomic number [13].

Broad usages of x-ray in different affairs necessitate specific occupational care by the personnel while at work whereas ionizing rays can make serious damages such as different cancers and chromosomal abnormalities as well as cataract, dermal damages, muscular and skeletal disorders. They can also damage thyroid gland and, nervous and reproductive systems [14]. Currently, there are a great number of personnel and passengers exposed to daily radiation released from the x-ray inspection devices in airports [15]. The x-ray damages will be prevented if proper mitigation and preventive strategies are adopted in airports [16]. Baggage x-ray inspection systems must be surveyed regularly. The monitoring frequency depends on the conditions of use, type of x-ray system and performance history. Anyhow, the monitoring frequency should be determined by the relevant authority. X-ray inspection systems must have adequate shielding against radiation to avoid operators or other individuals from being exposed to hazardous ambient radiation [17].

One of the most important measures in preventing radiation damages is continuous measuring of ambient x-ray [18] to keep it within the permissible limits [19]. The aim of this research was to investigate the x-ray exposure levels at inspection gates of two Airports, Mehrabad and Imam Khomeini.

## Materials and methods

This is a cross-sectional study done in autumn 2012 to measure the ambient x-ray exposure level at inspection gates of Mehrabad and Imam Khomeini Airports. There is a Flight Security Unit (FSU) separated as men and women gates in each airport responsible for inspecting passengers and their luggage. Like every other international airports, X-ray inspection systems are used in Mehrabad and Imam Khomeini Airports to inspect baggage of passengers. The FSU in the airports is equipped with inspection devices of RAPISCAN and HEIMANN types.

**Figure 1:**
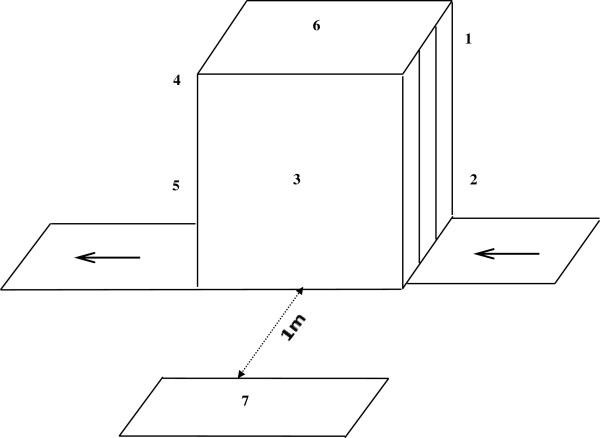
Position of measurement points at baggage x-ray machines.

Three operators work at each inspection gate including a line keeper who stands in front of the device exit, a rail operator up who release the luggage once engaged, and a computer operator who monitors the luggage status. In this study, the ambient X-Ray radiation of Mehrabad and Imam Khomeini airports were measured at two inspection gates separated by men and women entrances. All measurements were done by SMARTLON x-ray detector that is a proportional counter using the dosimetric technique to measure the absorbed dose of tissue equivalent according to International Atomic Energy Agency Standard, IAEA (Assessment of Occupational Exposure Due to External Sources of Radiation, Standards and manual, Series No. RS-G-1.3, 1999) [20,21]. The detector was calibrated by the manufacturing company according to Calibration of Radiation Protection Monitoring Instruments, Safety Reports Series No. 16, 2000 [22].In this research the ambient x-ray of the devices were measured at three positions of closed-curtain, open-curtain and beside the device. Nothing passes through the device at closed-curtain position as the protection curtain fully closes the exit door while in open-curtain position the luggage is exited from the device and the protection curtain is opened. At the position of “beside the device” the control operator seats nearby the device to monitor the luggage. Figure 1 shows measurement points of baggage x-ray machines at all positions.

Table 1 describes location of the X-Ray machines in both Mehrabad and Imam Khomeini Airports selected as dosimetry sampling in this study. As mentioned earlier, men and women enter into the waiting lobby of the airports from separated entrance gates.

**Table 1:** Location of the X-ray machines as the sampling points

**Measuring gates code**	**Mehrabad**			**Imam Khomeini**		
	**Terminal Location**	**Gate number**	**Gender (Men/Women)**	**Terminal location**	**Gate number**	**Gender (Men/Women)**
A	South	1	M	West	1	W
B	South	2	M	West	1	M
C	South	1	W	West	2	M
D	South	2	W	East	1	M
E	North	1	M	East	2	M
F	North	1	W	East	2	W

According the As Low as Reasonably Achievable (ALARA) principles recommended by International Commission on Radiological Protection (ICRP) for radiological protection, the collective equivalent dose of baggage x-ray inspection systems must be minimized as lowest as possible.

According to the recommendations by ACGIH in 2012, the allowable whole-body exposure to ionizing rays is 50 msv.h^-1^ per year (16). Since each year consists of 50 working weeks, the allowable limit of weekly exposure to x-ray (μsv.h^-1^) is calculated as Equivalent 1:

(1) $$\begin{array}{l} \text{Allowable}\;\text{Value}\;\text{of}\;\text{Weekly}\;\text{Exposure}\\ \;=\frac{\text{The}\;\text{allowable}\;\text{value}\;\text{per}\;\text{year}}{\text{number}\;\text{of}\;\text{working}\;\text{weeks}\;\text{per}\;\text{year}}\\ \;=\frac{50000}{50}=1000\;\text{μsv}.h^{‒1} \end{array}$$

Considering that each week include 40 working hours, the allowable hourly limit of exposure to x-ray (μsv.h^-1^) could be estimated through the Equation 2:

(2) $$\frac{\text{Allowable}\;\text{weekly}\;\text{dose}}{\text{working}\;\text{hours}\;\text{per}\;\text{week}}=\frac{1000}{40}=25\;\text{μsv}.h^{‒1}$$

## Results

The measurement results of ambient x-ray released from the devices RAPISCAN and HEIMANN at different positions are presented in Table 2.

**Table 2:** **Ambient x-ray radiation results by devices (μsv.h**^**−1**^**)**

**Devices**		**Allowable dose**	**Beside the device**	**Closed-curtain**	**Open-curtain**
RAPISCAN	Men	25	1.3	2	5
	Women	25	1.2	1.1	1.9
HEIMANN	Men	25	1.4	5.8	8.9
	Women	25	1.4	1.1	1.2

The results showed that the x-ray exposure level at all measurement points were lower than the allowable occupational dose of 25 μsv.h^-1^. In the location of the RAPISCAN device, the maximum and minimum exposure levels were respectively equal to 5 and 1.1 (μsv.h^-1^) when the device was in “open-curtain” position at men gate and in “closed-curtain” position at women gate (Table 2).

**Figure 2:**
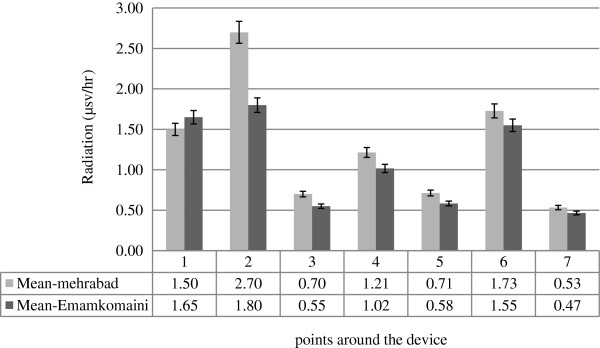
Comparison of radiation doses released from the devices in Mehrabad and Imam Khomeini Airports.

Additionally, the maximum and minimum exposure levels in the location of HEIMANN device were respectively equal to 8.9 and 1.1 μsv.h^-1^ when the device was in “open-curtain” position at men gate and in “closed-curtain” position at women gate.Figure 2 illustrates the average exposure levels at different points on the X-Ray device in various positions. In both airports, Mehrabad and Imam Khomeini, the maximum exposure level was detected at Point No. 2 (the entrance of inspection device); while a lower exposure level were measured at Point No. 3 (beside the devices) and also the minimum exposure level were at the point No. 7 where the operators check the baggage manually. The position of each measurement point is shown in Figure 1.

**Figure 3:**
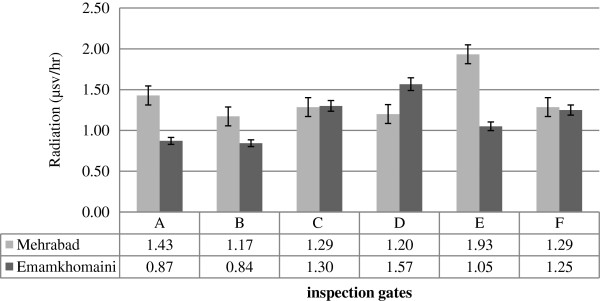
Comparison of radiation dose of measurement points surrounding the devices.

Figure 3 shows the comparison between the average x-ray radiation at different inspection gates in Mehrabad and Imam Khomeini Airports. As the figure suggests, the maximum x-ray rate in Mehrabad and Imam Khomeini Airports was measured at Gates D and E, respectively while Gate B contain the minimum x-ray leakage in both Mehrabad and Imam Khomeini Airports. The Location of each measurement point is shown in Table 1.

## Discussion

This study showed that the exposure level was lower than the occupational exposure limit at all measurement points. However, the permissible exposure level is substantially higher than that of Radiation Safety Institute of Canada presented the average annual dose of the baggage x-ray devices are less than 0.014 msv.h^-1^[23]. Additionally, an investigation by NIOSH at 12 airports in America revealed that the exposure dose at 90% of the stations were not measurable while the measured doses at remaining stations were lower than the Threshold Limit Value (TLV) [24].

Statistical test of “sign” shows a significant difference between the average x-ray exposure level (2.68 ± 0.73 μsv.h^-1^) and the standard dose of 25 μsv.h^-1^ (P ≤ 0.001). It means that the ambient x-ray is lower than the allowable occupational limit. The average x-ray exposure levels at different positions of open curtain, close-curtain and beside the devices were equal to 4.25 ± 1.75 μsv.h^-1^, 2.5 ± 1.12 μsv.h^-1^ and 1.3 ± 0.07 μsv.h^-1^, respectively. The measured values showed a statistically significant difference with the standard exposure levels (P ≤ 0.001). In other words, the ambient x-ray at inspection gates is lower than the standard limit.

The *t*-test results indicated that there is a significant difference between the x-ray exposure level at men and women gates (P = 0.015). The average x-ray exposure level at men and women gates were 4.07 ± 1.24 (μsv.h^-1^) and 1.3 ± 0.13 (μsv.h^-1^), respectively. This difference can be mainly due to the transportation of the largest packages at men gate and longer “open-curtain” position that cause releasing greater amount of radiation from the devices.

There no significant difference was found between the x-ray exposure level of devices RAPISCAN and HEIMANN (P = 0.699). It is worth mentioning that the average x-ray radiation from the devices RAPISCAN and HEIMANN were equal to 2.07 ± 0.61 μsv.h^-1^ and 3.3 ± 1.34 μsv.h^-1^, respectively. The measured values were both lower than the standard occupational limits. Based on the obtained results, the highest x-ray exposure (8.9 μsv.h^-1^) was measured in “open-curtain” position of the HEIMANN device at men inspection gate which is lower than the standard limit offered by ACCIH in 2012 [19].

According to a study by England et al. on similar devices, the x-ray exposure level was reported between 0–1 μsv.h^-1^ with no carcinogenic side effects [25]. Arnstein et al. showed that damaging effects of ionizing radiation is higher in people exposed constantly to X-rays for 8 hours [26]. The results of a similar studies done by Tanaka et al. [27] and NIOSH at the Airports Cincinnati, Baltimore, Boston, West Plan Beach, Providence and Miami, on x-ray devices of L3, TEX5500, and CTX2500 types it was revealed that the x-ray exposure levels of were lower than the allowable limits. Zhumadilov et al. reported similar results for the Japan Airport [28]. The above mentioned findings confirm the results of the present study.

## Conclusions

In the present study, low doses of ambient x-ray radiation were detected at inspection gates of Mehrabad and Imam Khomeini Airports. Although the measured X-ray is lower than the allowable limits, however, it cannot be regarded completely safe whereas the personnel are exposed to the radiation every day for 8 or even 12 hours. Accordingly, in order to protect the health of Flight Security Unit personnel and prevent them from being over-exposed to ionizing radiation, the use of personal protective equipment at workplace as well as adopting prevention and mitigation measures are of great importance.

As the results suggest, the highest radiation leakage was found at the entrance of the devices while the least leakage was measured beside the devices, at a distance of 1 m surrounding the devices. According to which, it can be concluded that operators receive a greater amount of radiation when standing in front of the devices and doing physical inspection. Therefore, it is recommended that they change their position and keep distance farther from 1 m surrounding the curtains of X-Ray Inspection Box.

## Competing interests

The authors declare that they have no competing interests.

## Authors’ contributions

GP participated in the design of the study and supervised the research progress based upon the schedule. FV did the statistical analyses. SN and AM wrote the initial draft of the paper and finalized it according to the other coauthors’ comments. All authors have read and approved the finalized manuscript.
